# Effective coverage practice in Ethiopia

**DOI:** 10.1136/bmjgh-2025-019105

**Published:** 2026-02-25

**Authors:** Seblewengel Lemma, Anene Tesfa Berhanu, Ashenif Tadele, Bantalem Yihun, Bereket Yakob, Dessalegn Y Melesse, Fikreselassie Getachew, Getachew Tollera, Hiwot Achamyeleh, Mihiretu Alemayehu Arba, Misrak Getnet, Joanna Schellenberg, Josephine Exley, Kassahun Alemu, Lars Åke Persson, Tadesse Guadu, Theodros Getachew, Zewditu Abdissa Denu, Zewdie Mullisa, Tanya Marchant, Effective coverage technical working group, Seblewengel Lemma

**Affiliations:** 1 Department of Disease Control, London School of Hygiene and Tropical Medicine, London, England, UK; 2 University College London Institute of Child Health, London, England, UK; 3 Health System and Reproductive Health Research Directorate, Ethiopian Public Health Institute, Addis Ababa, Ethiopia; 4 Public Health, Purdue University System, West Lafayette, Indiana, USA; 5 Ethiopia Ministry of Health, Addis Ababa, Addis Ababa, Ethiopia; 6 School of Nursing and Public Health, University of KwaZulu-Natal College of Health Sciences, Durban, KZN, South Africa; 7 School of Public Health, College of Health Sciences and Medicine, Wolaita Sodo University College, Sodo, SNNPR, Ethiopia; 8 Community Health Science, University of Manitoba, Winnipeg, Manitoba, Canada; 9 Office of the Director General, Ethiopian Public Health Institute, Addis Ababa, Ethiopia; 10 Department of Reproductive Health, College of Health Sciences and Medicine Wolaita Sodo University, Wolaita Sodo, Ethiopia; 11 University of Gondar College of Medicine and Health Sciences, Gondar, Ethiopia; 12 Department of Environmental Health, University of Gondar, Gondar, Ethiopia; 13 Global Health and Population, Harvard T H Chan School of Public Health, Boston, Massachusetts, USA; 14 Anaesthesia, University of Gondar College of Medicine and Health Sciences, Gondar, Amhara, Ethiopia; 15 Department of Environmental Health, University of Gondar College of Medicine and Health Sciences, Gondar, Amhara, Ethiopia; 16 John Snow Inc (JSI), Addis Ababa, Ethiopia

**Keywords:** Maternal health, Health systems, Health services research

## Abstract

Effective coverage measurement has emerged as a tool to help understand health system performance for the provision of high-quality health care. Using a cascade approach that combines data on demand- and supply-side steps, effective coverage measures highlight where gaps in the health system exist and how improvements might be made so that more people benefit from the potential of the health services available to them. In practice, however, there are challenges in making this work. This analysis paper aimed to highlight those challenges in calculating effective coverage in Ethiopia, using antenatal care as a test case, and propose a solution.

In Ethiopia, government leaders are committed to taking a data-informed approach to improving health care quality. To support this, an effective coverage technical working group was formed of individuals with experience of effective coverage analysis in Ethiopia to share knowledge and create learning for a way forward.

Through methods analysis of one common indicator, the effective coverage of antenatal care, four key challenges were identified by the group: (1) features of the data sources used, (2) the number of cascade steps included in the effective coverage calculations, (3) the data elements included within cascade steps and (4) the methods applied to generate composite indicators.

Multiple small differences were observed to have an influence on the usability of effective coverage measures for decision-making. The group concluded that greater transparency in reporting effective coverage measures was urgently needed and proposed and discussed the use of a reporting checklist for this purpose.

**Summary box**

- Effective coverage indicators that measure ‘the proportion of the target population in need of a health service who have a positive outcome as a result of that service’ are now recommended for measuring a country’s progress in providing high-quality healthcare services.
- A technical working group in Ethiopia has collaborated to share learning on how to operationalise effective coverage indicators in practice, identifying the need for more transparency in methods.
- Consistent use of an effective coverage indicator checklist is proposed. Transparent reporting of effective coverage methods will result in more harmonised indicator estimates between time and place, enhancing trust, interpretation and use of effective coverage for decision-making.

## Background

 Ethiopia has achieved major declines in maternal and child mortality and in the occurrence of common communicable diseases. Nonetheless, maternal and neonatal mortality remains high, and the growing prevalence of nutritional disorders, non-communicable diseases, injuries and mental illness constitutes a multiple burden of disease.^1^,^3^ To this end, the Ethiopian government has prioritised improvement in maternal, newborn and child healthcare and explicitly identified investments in healthcare quality and a health data revolution to underpin this goal.^3^,^5^ The Ministry of Health leadership in Ethiopia has identified effective coverage as an important tool to track progress in whether those needing a high-quality service receive it and have a positive outcome as a result.^6^ This means moving beyond indicators of service contact coverage to effective coverage measurement indicators that also reflect dimensions of healthcare quality, measured by working through a cascade of steps.

To support Ethiopia in this goal, in 2023, we established an informal effective coverage technical working group, bringing together actors from academia, policy and implementation for a regular monthly Zoom-based meeting to share learning about putting effective coverage indicators into practice. Members had the shared objectives of wanting to produce actionable effective coverage measures in Ethiopia, a preference to use existing national datasets rather than collecting new primary data and aligning effective coverage indicator definitions to the country setting.

Similar constraints and concerns in constructing effective coverage indicators in practice were shared and demonstrated in this commentary through three different national-level calculations of one indicator: effective coverage of antenatal care (ANC) (table 1).^7^,^9^ Of note is the challenge that, while all three calculations showed a consistent drop from contact coverage to effective coverage, differences in definitions and methods around quality adjustments resulted in different effective coverage estimates. Each calculation had used a different nationally representative dataset. These three datasets had different data elements available, resulting in differences in the number and definition of cascade steps. Two of the calculations used four ANC contacts as the starting point,^7 8^ while one used one ANC visit.^9^ Though each calculation used different methods, the effective coverage estimates were relatively close: the two estimates using four ANC contacts estimated effective coverage to be between 12% and 16%,^7 8^ while one estimate using one ANC contact estimated effective coverage to be 28%.^9^ Thus, this analysis paper aims to highlight the challenges experienced in calculating effective coverage in Ethiopia, using ANC as a test case, and to propose a standardised checklist to improve the transparency, comparability and usability of these metrics for decision-making.

**Table 1:** Effective coverage of ANC in Ethiopia: three examples using nationally representative datasets

Project	Data source	Reference period	Contact coverage	Input- adjusted coverage	Intervention- coverage	Process quality-adjusted coverage	Drivers of indicator definition
Using four ANC visits for contact coverage							
Lemma *et al*^20^	District Health Information System-2	2022–2023	48%	24%	16%	Not available in District Health Information Software-2	Government standards for provision of ANC mapped to available data elements
Abdissa *et al*^7^	Performance monitoring for action linked household and health facility survey	2019–2020	40%	28%	18%	12%	Government standards for provision of ANC mapped to availability of available data elements
Using one ANC visit for contact coverage							
Yakob *et al*^9^	Demographic and Health Survey linked to service provision assessment	2011–2016 service provision assessment: 2014	62%	29%	42%	22%	WHO guidelines for ANC mapped to available data elements

See online supplemental file 1 for details of the analysis.

ANC, antenatal care.

In the next section, we explore this experience in more detail.

## Issues arising in constructing effective coverage indicators

### Features of the data sources

Features of the data sources could all have an important influence on effective coverage estimates and should be reported. This analysis describes how data representativeness, data quality, linking and reference periods may drive variation in effective coverage estimates for Ethiopia.

#### Representativeness

Ethiopia has a routine health information system that captures data for all health facilities, digitally summarised using the open-source District Health Information Software-2 (DHIS-2).^10^ In addition, there are periodically conducted nationally representative household surveys such as the Demographic and Health Survey.^11^ There is also a periodic service provision assessment (SPA),^12^ a national sample survey that collects data from all levels of health facilities, and other programme or disease-specific surveys, including the performance monitoring for action (PMA),^13^ a linked national household and health facility survey.

Our three calculations were based on different data sources (the facility-based DHIS-2 only,^8^ the household and health facility-linked PMA^7^ and the household DHS linked with health facility SPA data).^9^ All three combinations were nationally representative of public sector ANC access and provision, meaning that the representativeness of our data source choices was not likely to have influenced differences between estimates.

Data completeness for individual items. This was particularly noted when working with the DHIS-2 data, as reported elsewhere.^14 15^ Some items indicative of quality were excluded from the analysis because of high levels of incompleteness, thus influencing healthcare quality definitions and likely creating a difference in the way each cascade step was defined.

#### Linking

For maternal, newborn and child health, no single national survey captures all required service contact and quality measures, and effective coverage indicators almost always require linking at least two datasets.^16^ The method of linking data could affect estimates.^17^

Yakob *et al* combined the population-level data from DHS 2016 with health facility-level information from SPA 2014 data; Abdisa *et al* used the linked PMA household and health facility survey data, and Lemma *et al* used DHIS-2 for all steps of the cascade except for estimating the target population (which was derived from the coverage of any ANC, as estimated from the recent DHS). All linking was carried out using the ecological method,^17^ meaning that differences in linking were unlikely to have been important drivers of differences between estimates.

#### Reference period

The data sources had different pregnancy reference periods. DHIS-2 pregnancies occurred between 2022 and 2023, PMA survey pregnancies occurred between 2019 and 2020, and the DHS data represented pregnancies between 2011 and 2016. Moreover, there was a gap of two or more years in the reference period for each facility data source. It is possible that these temporal gaps in the reference periods could explain the differences between the three calculations.

### Cascade steps included in the effective coverage calculation

**Figure 1:**
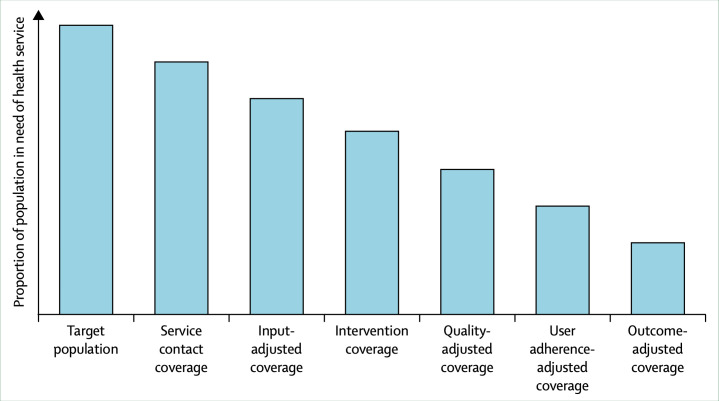
Standardised cascade for measuring effective coverage, reproduced from Marsh *et al*.^6^

As shown in figure 1, calculating effective coverage indicators involves working across a cascade with six possible steps. The completeness of the cascade depends on data availability to calculate each step in such a way that adequately reflects a shared conceptualisation of high-quality healthcare. For a complex service such as ANC, there is broad agreement that it is appropriate to go to the process quality-adjusted cascade step as a proxy measure for health service quality.^6^

The number of cascade steps applied was different between our three examples. While all three calculations reported an effective coverage measure for ANC, the availability of data elements in the chosen data source meant that one group reported only to the intervention-adjusted step (ie, calculating the proportion of women who attended services where inputs were available and interventions such as syphilis testing were provided).^8^ Two calculations were able to estimate process quality-adjusted coverage (ie, calculating the proportion of women who attended services where inputs were available, interventions were in place and health workers followed appropriate standards).^7 9^ Differences in the number of cascade steps included partially explain the variability between our estimates.^6^

### Data elements included within cascade steps

Currently, there is no standard guidance on the definition of individual cascade steps. One consequence of this is that definitions vary between examples, as evident in the systematic literature review of effective coverage indicators.^18^

There was limited consistency in the definition of cascade steps between our groups. Even in the contact coverage step, one calculation defined contact coverage as at least one ANC contact, and two used at least four contacts. For the definition of the quality-adjusted steps, all groups tried to follow ANC guidelines. Ultimately, the main driver was the data elements available (eg, which drugs had been recorded in facility readiness inventories or which health worker behaviours had been observed in surveys), meaning that different proxies for quality were applied between groups. A second driver affecting the definition of individual steps was the awareness that if too many data elements were included in a given step, then the output would be too complex, overly negative and not actionable. The Technical Working Group discussed at length the difficult decisions made regarding whether or not to include data elements that seemed important proxies for quality but that resulted in estimates of zero ANC coverage, an outcome unlikely to be acceptable to policymakers.

Differences in the data elements included within cascade steps partially explain the differences between our estimates.

### Methods for generating composite indicators

Additional complexity in effective coverage measurement lies in the methods used to combine multiple data elements in each cascade step to create composite indicators.

In the example of ANC, input-adjusted coverage requires a composite indicator that reflects adequate facility infrastructure, adequate staffing and availability of multiple drugs or commodities. Whether and how to weigh these elements adds another layer of complexity. The method of combining data elements influences the output, whether calculating a score, a binary success-failure variable or using threshold levels.^19^

Our calculations all used different methods to generate composite indicators within cascade steps. For the input-adjusted coverage, two calculations^7 9^ used a percentage-based composite score, while one^8^ used a combination of threshold approach and all-or-none scoring. For the intervention and process-adjusted coverages, Abdisa *et al* used all-or-none scoring; Lemma *et al* used indicator averages, and Yakob *et al* used a percentage-based composite score to generate the quality index. In all the calculations, each item was given equal weight in the respective cascade steps.

It is likely that differences in methods to generate composite indicators also contributed to differences between estimates.

## Implications for implementation and measurement practice

Irrespective of the differences in the construction of the individual indicators demonstrated here, it is consistently clear that a quality gap exists between ANC contact coverage and the effective coverage of ANC in Ethiopia, and this problem must be addressed.

The real utility of the effective coverage measure lies in understanding the shape of the drop-off across the cascade steps: examining where coverage is lost and where bottlenecks in service provision exist can help programmes identify the biggest problems and plan actions accordingly. Nonetheless, the indicator needs to be trusted for decision-making, and this trust will remain suboptimal without further measurement guidance.^20^ Characteristics that help to make indicators trusted by decision-making include that they^1^ have clear public health importance,^2^ are feasible to collect,^3^ produce timely results with action implications,^4^ reflect accurate measures and^5^ have consistency between time and place, permitting the tracking of trends.^21^ While the first three characteristics are broadly present in our effective coverage examples from Ethiopia, the different definitions and calculation methods mean that the fourth and fifth characteristics are currently missing.

**Figure 2:**
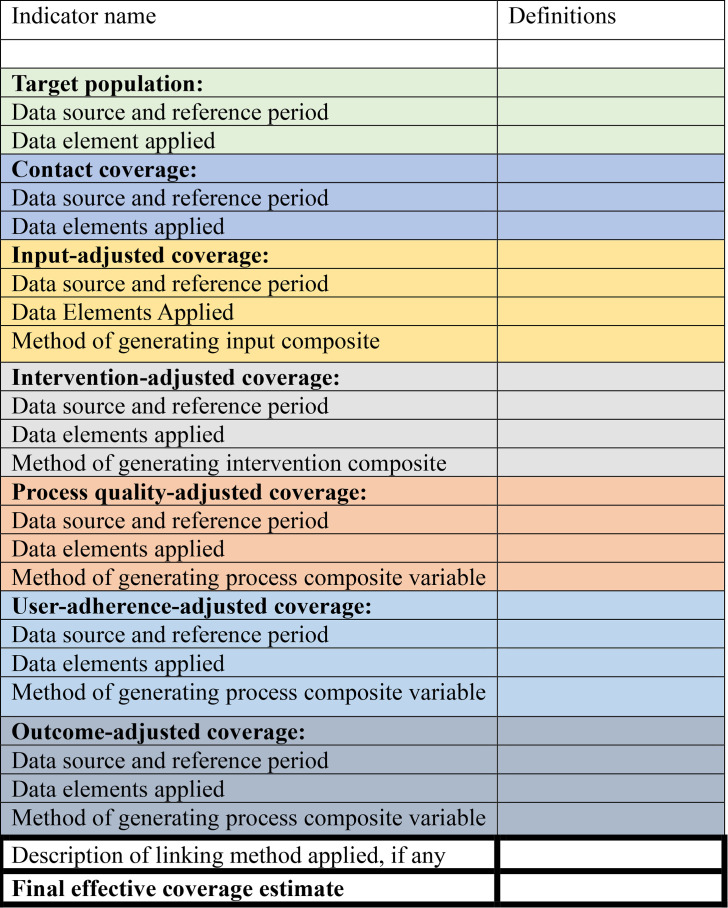
Checklist for the transparent reporting of effective coverage indicators.

We recommend the use of an effective coverage indicator checklist by all actors reporting effective coverage results (figure 2) at any level of the health system, as the level of granularity needed differs across levels of the health system. We illustrate how this checklist can be used to promote transparent reporting by completing it for the effective coverage examples discussed in this paper at the national level (online supplemental tables 1-3).

The checklist is organised by cascade step to reflect five characteristics: (1) the data sources, (2) the reference periods, (3) the precise definition of each step, (4) the methods applied in creating composite variables for each step and (5) the linking methods applied where more than one data source is used. Applying this indicator checklist to our examples in Ethiopia helped to identify the challenges discussed above and permitted a more nuanced interpretation of the output.

The use of the checklist makes the information accessible for researchers and technicians who look for examples of effective coverage in the literature to develop their own. It also enhances the use of standards and transparency in reporting results that further improve trustworthiness and comparability of results within and across countries.

This checklist has the potential to be useful in any context beyond Ethiopia (academia, programme and government) as it is not attached to or influenced by any contextual factors and follows the globally recommended effective coverage cascade concept and approach.^6^

## Conclusion

Many countries see the potential use of effective coverage measurement to inform health system decision-making. However, in the absence of gold-standard harmonised tabulation plans for effective coverage indicators, the outputs currently in circulation produce divergent estimates that limit trust in their use for decision-making. Creating such fixed tabulation plans is a challenge because effective coverage indicators need to reflect country contexts, and national datasets include different data elements on healthcare quality. If decision-makers want to use effective coverage indicators for tracking progress or benchmarking between time and place, then country-led measurement investment and expertise will be needed to advance the situation.^22^

To improve the situation now, the effective coverage technical working group in Ethiopia suggests the use of an indicator checklist. Taken together with more guidance on methods and national definitions of service packages, the consistent use of this checklist has the potential to advance the effective coverage measurement agenda in Ethiopia and beyond and, in doing so, to better serve a national and global public health need.

## Supplementary material

10.1136/bmjgh-2025-019105 online supplemental file 1

## Data Availability

All data relevant to the study are included in the article.

## References

[1] Ruducha J, Mann C, Singh NS (2017). How Ethiopia achieved Millennium Development Goal 4 through multisectoral interventions: a Countdown to 2015 case study. Lancet Glob Health.

[2] Mehretie Adinew Y, Feleke SA, Mengesha ZB (2017). Childhood Mortality: Trends and Determinants in Ethiopia from 1990 to 2015—A Systematic Review. Adv Public Health.

[3] Ministry of Health (2021). Health. Health Sector Transformation Plan II (HSTP II) 2020/21 - 2024/25 (2013 EFY-2017 EFY). http://repository.iphce.org/xmlui/handle/123456789/1414.

[4] (2024). National health care quality and safty strategy (2021-2025). https://pdf.usaid.gov/pdf_docs/PA00ZS5J.pdf.

[5] Ministry of Health (2017). National MNH Quality of Care Roadmap (2017/18-2019/2020). http://repository.iphce.org/xmlui/handle/123456789/738.

[6] Marsh AD, Muzigaba M, Diaz T (2020). Effective coverage measurement in maternal, newborn, child, and adolescent health and nutrition: progress, future prospects, and implications for quality health systems. Lancet Glob Health.

[7] Abdissa Z, Alemu K, Lemma S (2024). Effective coverage of antenatal care services in Ethiopia: a population-based cross-sectional study. BMC Pregnancy Childbirth.

[8] Lemma S, Getachew F, Achamyeleh H (2026). Effective coverage of maternal, neonatal and child health services based on District Health Information System 2 (DHIS2) data in Ethiopia: a mixed-methods study. BMJ Open.

[9] Yakob B, Gage A, Nigatu TG (2019). Low effective coverage of family planning and antenatal care services in Ethiopia. Int J Qual Health Care.

[10] DHIS2 (2024). MINISTRY of health - Ethiopia. https://www.moh.gov.et/en/projects-3-col/dhis2?language_content_entity=en.

[11] Central Statistics Authority, ICF (2017). Ethiopia demographic and health survey 2016. https://dhsprogram.com/publications/publication-fr328-dhs-final-reports.cfm.

[12] Institute EPH, Health EM of, ICF (2023). Ethiopia service provision assessment 2021–22. https://dhsprogram.com/publications/publication-SPA36-SPA-Final-Reports.cfm.

[13] Zimmerman L, Desta S, Yihdego M (2020). Protocol for PMA-Ethiopia: A new data source for cross-sectional and longitudinal data of reproductive, maternal, and newborn health. Gates Open Res.

[14] Adane A, Adege TM, Ahmed MM (2021). Routine health management information system data in Ethiopia: consistency, trends, and challenges. Glob Health Action.

[15] Bhattacharya AA, Allen E, Umar N (2020). Improving the quality of routine maternal and newborn data captured in primary health facilities in Gombe State, Northeastern Nigeria: a before-and-after study. BMJ Open.

[16] Munos MK, Stanton CK, Bryce J (2017). Improving coverage measurement for reproductive, maternal, neonatal and child health: gaps and opportunities. J Glob Health.

[17] Carter ED, Leslie HH, Marchant T (2021). Methodological considerations for linking household and healthcare provider data for estimating effective coverage: a systematic review. BMJ Open.

[18] Exley J, Gupta PA, Schellenberg J (2022). A rapid systematic review and evidence synthesis of effective coverage measures and cascades for childbirth, newborn and child health in low- and middle-income countries. J Glob Health.

[19] Kara P, Valentin JB, Mainz J (2022). Composite measures of quality of health care: Evidence mapping of methodology and reporting. PLoS ONE.

[20] Lemma S, Tesfa A, Getachew F (2024). Operationalising effective coverage measurement in Ethiopia: a qualitative study. J Glob Health Rep.

[21] Marchant T, Bryce J, Victora C (2016). Improved measurement for mothers, newborns and children in the era of the Sustainable Development Goals. J Glob Health.

[22] Strong K, Konstantinou G, Agweyu A (2024). Recommendations for Using Health Service Coverage Cascades to Measure Effective Coverage for Maternal, Newborn, Child, and Adolescent Health Services or Interventions. Glob Health Sci Pract.

