# Supplementary material for: Effective coverage practice in Ethiopia
Source: BMJ Glob Health. 2026 Feb 25;11(2):e019105. doi: 10.1136/bmjgh-2025-019105 (PMC12970043; doi:10.1136/bmjgh-2025-019105)
Supplement: online supplemental file 1 [file bmjgh-11-2-s001.docx]

**Supplementary tables: Effective coverage of antenatal care in Ethiopia: example calculations using different nationally representative data sets**

**Table S1. Effective coverage of antenatal care in Ethiopia: calculated using the nationally-representative data set District Health Information System-2 ^1^**

| **Indicator name** | **Effective coverage of antenatal care (4 visits)** |  |
| --- | --- | --- |
| **Definition of the indicator** | % of the population in need of quality antenatal care that received at least four antenatal care visits with quality |  |
| **Target population:** | **All pregnant mothers** | **100%** |
| *Data source and reference period* | DHIS-2, from July 2022 to June 2023 |  |
| *Data element applied* | Number of women in the target population |  |
| **Contact coverage:** | % of pregnant mothers who came to the health facility for their 4^th^ ANC service | **48%** |
| *Data source and reference period* | DHIS-2 from July 2022 to June 2023 |  |
| *Data elements applied* | Total number of pregnancies by region; total number of ANC4 visits |  |
| **Input-adjusted coverage:** | **% of mothers who seek for their 4^th^ ANC in a health facility that is ready to provide the service.** | **24%** |
| *Data source and reference period* | DHIS2 and 2022-2023 |  |
| *Data elements applied* | **Infrastructure***:* Presence of electricity, water, and sanitation facility |  |
|  | **Essential medicine***:* Availability of 23 essential tracer drugs according to government list of essential drug (Iron folate, Amoxicillin, magnesium sulphate injection, normal saline09, glucose, Adrenaline injection, Amlodipine tablet, Arthmeter-Lumfanthrine, Cipro-floxacillin, Cotrimoxazole240mg5ml, Frusemide tablets, Gentamycin injection, Hydralazine injection, Metformin tablet, Metronidazole capsule, ORS+/-Zinc sulphate, Omeprazole capsule, Oxytocin injection, Pentavalent vaccine, RHZE/RH, TDF/3TC/DTG, TTC eye ointment, Tetanus Anti-Toxin (TAT)) |  |
|  | **Human resource:** # of staff at the beginning of the year: nurse, midwife, health officer |  |
| *Other category (specify)* | None |  |
| *Method of generating input composite* | **Health Infrastructure:** percentage of facilities had at least one item; **Staffing**: % of facilities having average and above number of health workers and **Tracer drugs**: % of facilities with average and above percent of tracer drugs |  |
| **Intervention-adjusted coverage:** |  | **16%** |
| *Data source and reference period* | DHIS2 from July 2022 to June 2023 |  |
| *Data elements applied* | Tested for hepatitis B, tested for syphilis, received at least one TD vaccine and received iron folate |  |
| *Method of generating intervention composite* | Average of the percentages of the four interventions (tested for hepatitis B, for syphilis, received at least one TD vaccine and received iron folate) |  |
| **Process quality-adjusted coverage:** | **Not available** | **n/a** |
| *Data source and reference period* | n/a |  |
| *Data elements applied* | n/a |  |
| *Method of generating process composite* | n/a |  |
| **User-adherence-adjusted coverage:** | **Not relevant** | **n/a** |
| *Data source and reference period* | n/a |  |
| *Data elements applied* | n/a |  |
| *Method of generating process composite* | n/a |  |
| **Outcome-adjusted coverage:** | **Not relevant** | **n/a** |
| *Data source and reference period* | n/a |  |
| *Data elements applied* | n/a |  |
| *Method of generating process composite* | n/a |  |
| **Description of linking method applied, if any** | We used ecological linking to link the input data with aggregated mothers data. |  |
| **Final effective coverage estimate** |  | **16%** |

^1^Further information see Lemma S, Tesfa A, Getachew F, et al. Operationalising effective coverage measurement in Ethiopia: a qualitative study. *Journal of Global Health Reports*. 2024;8:e2024011. [doi:10.29392/001c.94935](https://doi.org/10.29392/001c.94935)

**Table S2. Effective coverage of antenatal care in Ethiopia: calculated using the nationally-representative Performance Monitoring for Action linked household and health facility data set ^2^**

| **Indicator name** | **Effective coverage of antenatal care (4 visits)** |  |
| --- | --- | --- |
| **Definition of the indicator** | % of the population in need of quality antenatal care that received at least four antenatal care visits with quality |  |
| **Target population:** |  | **100%** |
| *Data source and reference period* | PMA data, from 2019 to 2020 |  |
| *Data element applied* | Number of women aged 15-49 years in the study population |  |
| **Contact coverage:** |  | **40%** |
| *Data source and reference period* | PMA data, from 2019 to 2020 |  |
| *Data elements applied* | Total number of women received four or more ANC |  |
| **Input-adjusted coverage:** |  | **28%** |
| *Data source and reference period* | PMA women data, 2019-2020 & PMA service delivery assessment data 2019 |  |
| *Data elements applied* | **Infrastructure:** Presence of emergency transport, ANC room (2 items) |  |
|  | **Equipment:** Blood pressure apparatus, Fetoscope, weighing scale (3 items) |  |
|  | **Essential medicines:** Iron folate, Tetanus vaccination (2 items) |  |
|  | **Human resource:** None |  |
|  | **Diagnostic capacity:** haemoglobin test, blood glucose test, blood group test, VDRL test, Urine protein test (Urine dipstick) (5 items) |  |
| *Method of generating input composite* | Adding all yes responses and dividing by 12 (number of tracer items). The average availability score was computed and used to compute input-adjusted ANC coverage |  |
| **Intervention-adjusted coverage:** |  | **18%** |
| *Data source and reference period* | PMA women data 2019-2020 |  |
| *Data elements applied* | Percentage of ANC4 users who were given Iron folate tablets and tetanus vaccination |  |
| *Method of generating intervention composite* | Multiplication of the percentages |  |
| **Process quality-adjusted coverage:** | Using 9 process quality components based on national ANC guideline | **12%** |
| *Data source and reference period* | PMA women data 2019-2020 |  |
| *Data elements applied* | Blood pressure measured, Weight taken, Blood sample taken, Urine sample taken, Provider discussed healthy diet, Provider discussed where to go for delivery, Provider discussed transport for delivery, Provider discussed dangers of bleeding before delivery, Provider discussed dangers of high blood pressure |  |
| *Method of generating process composite* | Adding all yes responses and dividing by 9 (number of tracer items) |  |
| **User-adherence-adjusted coverage:** | Not applied | **n/a** |
| *Data source and reference period* | n/a |  |
| *Data elements applied* | n/a |  |
| *Method of generating process composite* | n/a |  |
| **Outcome-adjusted coverage:** | **Not relevant** | **n/a** |
| *Data source and reference period* | n/a |  |
| *Data elements applied* | n/a |  |
| *Method of generating process composite* | n/a |  |
| **Description of linking method applied, if any** | We used ecological linking method with the assumption women visited one of the health facilities in their catchment area |  |
| **Final effective coverage estimate** |  | **12%** |

^2^Further information see Abdissa Z, Alemu K, Lemma S, *et. al.* Effective coverage of antenatal care services in Ethiopia: a population-based cross-sectional study. BMC Pregnancy Childbirth. 2024 Apr 27;24(1):330. doi: 10.1186/s12884-024-06536-6.

**Table S3. Effective coverage of antenatal care in Ethiopia: calculated using the nationally-representative Demographic & Health Survey linked to Service Provision Assessment dataset ^3^**

| **Indicator name** | **Effective coverage of first antenatal care in Ethiopia, 2014** |  |
| --- | --- | --- |
| **Definition of the indicator** | % of pregnant women received quality antenatal care (where the care providers adhered to the standard clinical actions) |  |
| **Target population:** |  | **100%** |
| *Data source and reference period* | SPA+2014  DHS 2016 (2011 – 2016) |  |
| *Data element applied* | Number of women in the target population (DHS 2016)  Number of women receiving ANC observed (SPA+2014) |  |
| **Contact coverage:** |  | **62%** |
| *Data source and reference period* | DHS 2016 (2011 – 2016) |  |
| *Data elements applied* | Number of women in the target population receiving at least one ANC (DHS 2016) |  |
| **Input-adjusted coverage:** |  | **29%** |
| *Data source and reference period* | SPA+2014 |  |
| *Data elements applied* | **Infrastructure:** electricity, water, toilet, privacy, communication facility, and ambulance (6 items)  **Basic Equipment:** visual aid, BP apparatus, stethoscope, examination light, fetoscope, adult scale, examination bed, tape for fundal height, and ANC patient cards (9 items)  **Infection prevention:** sharps disposal system, hazardous substance disposal system, soap, water, hand disinfection solutions, waste bin, sterile syringes, surface disinfectants, gloves, and hand cleaning substances (10 items)  **Diagnostic capacity:** haemoglobin, blood glucose, malaria lab test, urine protein test, urine glucose test, HIV test, syphilis test, and pregnancy test (8 items)  **Essential medicines:** iron, folic acid, tetanus toxoid, ART, and amoxicillin (5 items)  **Human resource**: staff trained on ANC-related topics in the last 24 months (2 items)  **Guidelines:** ANC guidelines, other ANC-related guidelines, and infection prevention guidelines (3 items) |  |
| *Method of generating input composite* | First: The general service readiness index created for facilities having the required items, amenities, and capacities for ANC 1  Second: The general service readiness index was multiplied by the contact coverage |  |
| **Intervention-adjusted coverage:** |  | **42%** |
| *Data source and reference period* | SPA+ 2014 |  |
| *Data elements applied* | - % of pregnant women coming for ANC1 observed receiving iron folate and TT injection |  |
| *Method of generating intervention composite* | Created ANC 1 interventions index  Multiplying contact coverage by ANC 1 interventions index |  |
| **Process quality-adjusted coverage:** |  | **22%** |
| *Data source and reference period* | SPA+2014 |  |
| *Data elements applied* | 59 ANC 1 clinical actions per the WHO guidelines considered, i.e.   - Past pregnancy history (11 items) - Asking danger signs of current pregnancy (6 items) - Conducting physical examination and vital signs (10 items) - Conducting routine lab tests and investigations (5 items) - Providing routine supplements (2 items) - Counselling danger signs during pregnancy (7 items) - Counselling healthy pregnancy (8 items) - Natal preparation and postnatal care counselling (10 items) |  |
| *Method of generating process composite* | Index created from 59 items, then multiplied by contact coverage |  |
| **User-adherence-adjusted coverage:** | **n/a** | **n/a** |
| *Data source and reference period* | n/a |  |
| *Data elements applied* | n/a |  |
| *Method of generating process composite* | n/a |  |
| **Outcome-adjusted coverage:** | **n/a** | **n/a** |
| *Data source and reference period* | n/a |  |
| *Data elements applied* | n/a |  |
| *Method of generating process composite* | n/a |  |
| **Description of linking method applied if any** |  |  |

^3.^ For further information see Yakob B, Gage A, Nigatu TG, *et. al.* Low effective coverage of family planning and antenatal care services in Ethiopia. Int J Qual Health Care. 2019 Dec 31;31(10):725-732. doi: 10.1093/intqhc/mzy251.
